# Construction and validation of a clinical prediction model for asymptomatic obstructive coronary stenosis in patients with carotid stenosis

**DOI:** 10.3389/fcvm.2023.1096020

**Published:** 2023-09-07

**Authors:** Cuijie Qin, Chuang Li, Yunpeng Luo, Zhen Li, Hui Cao

**Affiliations:** Department of Endovascular Surgery, The First Affiliated Hospital of Zhengzhou University, Zhengzhou University, Zhengzhou, China

**Keywords:** carotid stenosis, coronary stenosis, prediction model, nomogram, LASSO regression analysis

## Abstract

**Background:**

Coronary artery stenosis occurs frequently in patients with carotid artery stenosis. We developed a clinical predictive model to investigate the clinical risk of asymptomatic obstructive coronary artery stenosis in patients with carotid artery stenosis ≥ 50%.

**Methods:**

From January 2018 to January 2022, carotid stenosis patients hospitalized at the First Affiliated Hospital of Zhengzhou University's Department of Endovascular Surgery were subjected to a retrospective analysis of their clinical information and imaging results. Excluded criteria were patients with lacking data, symptomatic coronary stenosis, prior coronary artery bypass grafting, and coronary stent implantation. Patients were separated into case and control groups according to whether or not they had obstructive coronary stenosis. Independent predictors were screened using univariate and multivariate logistic regression, and their accuracy was confirmed using least absolute shrinkage and selection operator (LASSO) regression. A Nomogram prediction model was developed using the aforementioned filtered factors. The model's discrimination and specificity were evaluated using the receiver operating characteristic curve (ROC) and Hosmer-Lemeshow goodness-of-fit test. Internal validation employed the Bootstrap procedure. The clinical decision curve analysis (DCA) of the prediction model was developed to assess the clinical applicability of the model.

**Results:**

The investigation included a total of 227 patients, of whom 132 (58.1%) had coronary artery stenosis. Hypertension, Grade I plaque, HbA1c ≥ 7.0%, MPV ≥ 9.2fl, and Fib ≥ 3.0 g/L were independent predictors, with OR values of (2.506, 0.219, 0.457, 1.876, 2.005), according to multivariate logistic regression. Risk factor screening and validation using lasso regression. The predictors chosen based on the optimal *λ* value are consistent with the predictors identified by multiple regression. The area under the ROC curve (AUC) of the model based on the above predictors was 0.701 (0.633–0.770), indicating that the model discriminated well. The calibration curve of the model closely matched the actual curve, and *P* > 0.05 in the Hosmer-Lemeshow goodness-of-fit test indicated the model's accuracy. The results of the DCA curve demonstrate the clinical applicability of the prediction model.

**Conclusion:**

Hypertension, grade I plaque, HbA1c ≥ 7.0%, MPV ≥ 9.2 fl, and Fib ≥ 3.0 g/L are predictors of asymptomatic coronary stenosis in patients with carotid stenosis ≥50%. The diagnostic model is clinically applicable and useful for identifying patients at high risk.

## Introduction

Stroke has an elevated level of morbidity and mortality and has become a serious hazard to the health of the elderly. According to accounts, carotid stenosis causes 25% to 30% of ischemic strokes (1). Atherosclerosis is the principal cause of carotid stenosis. Stenosis occurs most frequently at the bifurcation of the common carotid, the origin of the internal carotid, and the siphon (2). Effective early treatment of carotid stenosis can reduce the incidence of ischemic stroke. Central interventions included endarterectomy of the carotid artery, stenting of the carotid artery, and drug therapy (3). Atherosclerosis has a variety of effects and can accumulate in both large and medium arteries. Patients with carotid artery stenosis and coronary artery stenosis are not uncommon. Research have demonstrated that patients with both carotid artery stenosis and coronary artery stenosis have a substantially increased risk of acute cardiovascular disease during surgery (4). Compared to symptomatic coronary stenosis, asymptomatic coronary stenosis has an elevated likelihood of misdiagnosis due to the absence of typical symptoms. A number of studies have investigated accurate predictors of asymptomatic coronary artery stenosis in patients. A retrospective study revealed that the thickness, extent, and complexity of aortic arch lesions were independent risk factors for asymptomatic coronary stenosis greater than 50% (5). In addition, patients with insulin resistance are more likely to have asymptomatic coronary artery stenosis ≥ 50% and have a positive correlation with the number and severity of coronary artery stenosis (6). The purpose of this study was to retrospectively analyze the clinical data of patients with carotid artery stenosis ≥ 50% in our center, to investigate the related predictive factors of asymptomatic obstructive coronary stenosis, and to establish a nomogram prediction model in order to provide support for early detection of occult coronary disease in patients with carotid stenosis.

## Materials and methods

### Patients and data collection

**Figure 1:**
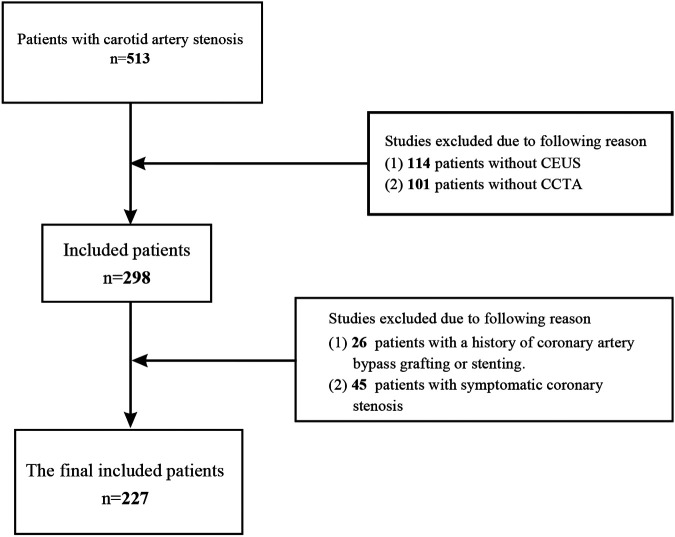
The flow chart of this study.

This survey included carotid artery stenosis patients admitted to the Department of Endovascular Surgery at the First Affiliated Hospital of Zhengzhou University between January 2018 and January 2022. By carotid ultrasound and carotid computed tomography angiography, a total of 513 patients with unilateral or bilateral carotid stenosis ≥ 50% were identified. Exclusion criteria included 45 patients with symptomatic coronary artery stenosis, 26 patients with a history of coronary stent implantation or coronary artery bypass grafting, 114 patients without carotid contrast-enhanced ultrasonography (CEUS), and 101 patients without coronary angiography. Finally, 227 patients in total met the inclusion criteria. They were split into a case group and a control group based on whether the degree of coronary artery stenosis exceeded 50%. Figure 1 shows the study's flowchart. The study was approved by the local ethics committee and was conducted in accordance with the 1964 Declaration of Helsinki.

The electronic medical record system was queried to obtain baseline clinical data, laboratory tests, and imaging investigations. Patient characteristics including age, gender, BMI, diabetes mellitus (DM), essential hypertension, and smoking and alcohol history. Laboratory examination indexes including hemoglobin A1c (HbA1c), hematocrit (HCT), lymphocyte percentage (LY%), monocyte percentage (MONO%), platelet count (PLT), mean platelet volume (MPV), albumin (Alb), fibrinogen (Fib), β2-MG, total cholesterol (TC), triglycerides (TG), high density lipoprotein (HDL), low density lipoprotein (LDL), lipoprotein(a) [Lp(a)], glomerular filtration rate (GFR), creatinine (Cr), homocysteine (Hcy). Imaging results including sum (PS) and maximum (P-max) of the plaque thickness, the degree of carotid artery and coronary artery stenosis and the carotid plaque level.

Hypertension was defined as blood pressure > 140/90 mmHg or treatments with antihypertensive medication. The diagnosis of diabetes mellitus was based on the 2017 Standards of Medical Care in Diabetes (7). In coronary computed tomography angiography (CCTA), the degree of coronary stenosis was classified as obstructive (luminal stenosis ≥ 50% of the coronary diameter) or non-obstructive (luminal stenosis < 50%) (8). Multi-site stenosis of a single vessel is determined by the most severe stenosis, and multi-vessel stenosis is defined as stenosis of two or more vessels (9). Guidelines for the diagnosis and treatment of carotid stenosis were used to classify symptomatic carotid stenosis (SCS) and asymptomatic carotid stenosis (3). A current smoker is defined as someone who has smoked cigarettes on a regular basis within the past six months, whereas an alcoholic is defined as someone who has consumed alcohol on a regular basis for over a year. The BMI was computed by dividing the weight (kg) by the square of the height (m). P-max was measured as the vertical distance between the tip of the plaque and adventitia interface of the lumen. PS was the sum of bilateral maximal carotid plaque thickness. The PS and P-max were determined by carotid artery ultrasound (10). Using the CEUS plaque blood flow grading standard, the grade of carotid plaque was classified as grades I to IV. Grade I was defined as the absence of plaque enhancement, grade II as the presence of one to three punctate plaque enhancements, and grade III as the presence of more than three punctate plaque enhancements or one to two short-line plaque enhancements. Grade IV is characterised by the presence of two or more linear enhancements in the plaque that are penetrating or predominantly penetrating the plaque, or by a sense of flow (11).

### Statistical analysis

Data analysis was performed using SPSS 23.0 and R version 4.2.1. Except for age, BMI, and HbA1c, the mean and median were chosen as the boundary values for the continuous variables of normal distribution and non-normal distribution, and the data were converted to binary variables for subsequent analysis. Multiple logistic regression included the binary logistic regression analysis factors with *P* < 0.05. Using backward stepwise multiple logistic analysis, independent predictors were identified. Selected predictors were cross-validated further utilising LASSO regression. The L1-penalized LASSO regression was applied to reduce the data dimensionality to avoid potential collinearity and overfitting among variables. The best lambda value was selected in LASSO regression using tenfold cross-validation. Under the lambda compression (lambda.1se), the variables with small regression coefficients were directly compressed to 0 to eliminate the corresponding variables. Using the “rms” programme, statistically significant factors were used to construct the nomogram and generate a prediction model. To evaluate the accuracy of the prediction model, we calculated the consistency index (C-index) using the “rms” programme. By constructing a calibration curve, the level of calibration was determined. The greater the consistency of the prediction model, the closer the calibration curve of the model to the standard curve. Using DCA, the clinical utility of the nomogram was determined by quantifying the net benefits of the probability of various threshold values in the array. Finally, an internal validation approach was utilised to evaluate the prediction model's stability.

## Results

### Patient characteristics

This study included 227 patients, involving 181 males (79.7%) and 46 females (20.3%). Subjects were divided into case and control groups according to whether they had obstructive coronary stenosis simultaneously. There were 132 cases in the case group (58.1%) and 95 cases in the control group (41.9%). There were 62 cases (47.0%) of single-vessel disease and 70 cases (53.0%) of multi-vessel disease in the case group. All patients exhibited no coronary stenosis-related symptoms.

### Analysis of predictive factors

**Figure 2:**
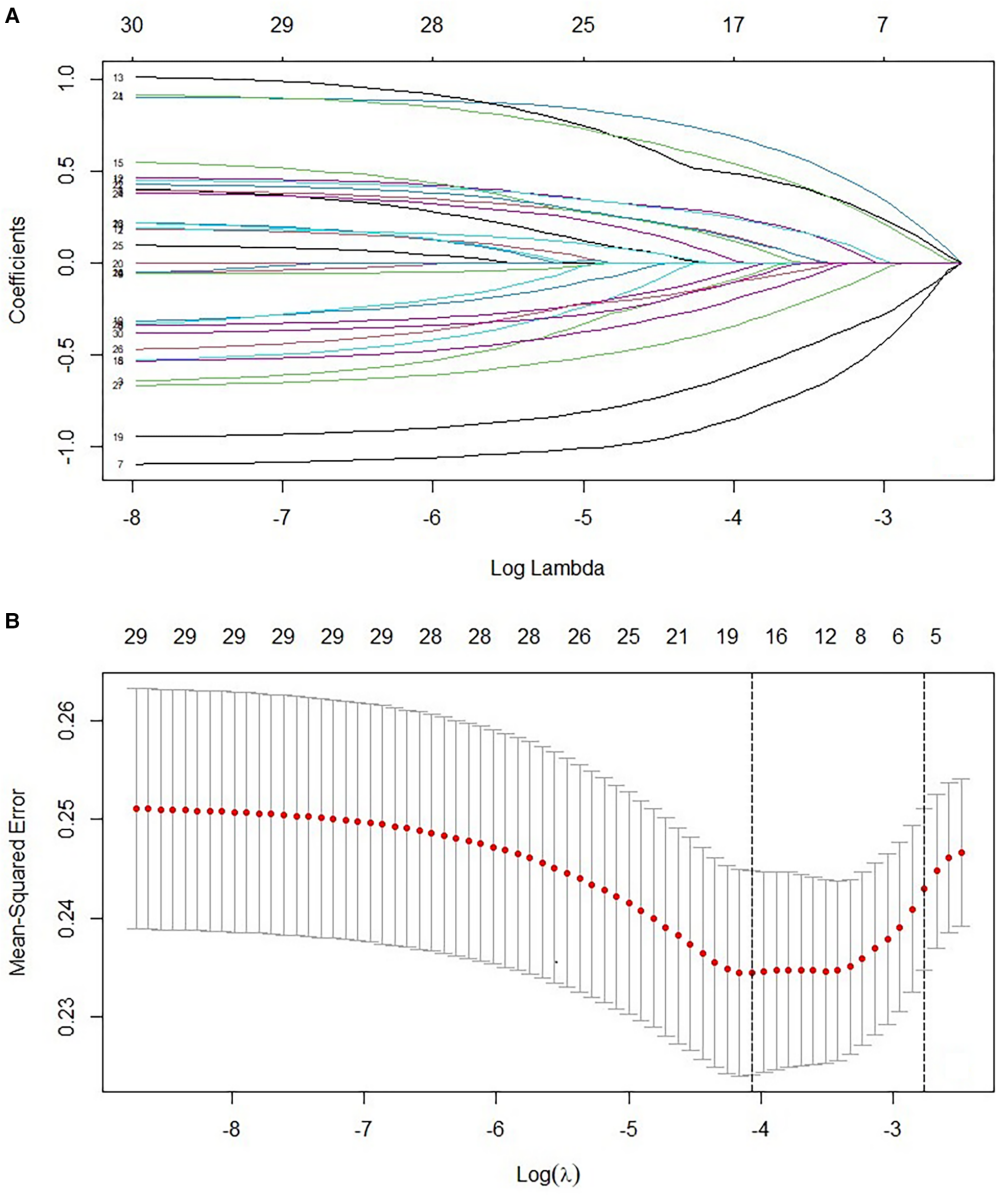
(**A**) LASSO coefficient profiles of 227 patients with obstructive coronary stenosis. A coefficient profile plot was produced against the log (λ) sequence. (**B**) Texture feature selection by using the least absolute shrinkage and selection operator (LASSO) binary logistic regression model. Tuning parameter (λ) selection in the LASSO model used 10-fold cross-validation via maximum AUC. A λ value of 0.0629 was chosen (1-SE criteria) according to 10-fold cross-validation.

Thirty laboratory parameters and imaging variables were included as independent variables in the binary logistic regression (Table 1). Hypertension, Grade I plaque, HbA1c, MPV, and Fib were statistically significant predictors (*P* < 0.05) based on the results of binary logistic regression. Binary logistic regression showed that Hypertension, Grade I plaque, HbA1c, MPV, and Fib were statistically significant predictors (*P* < 0.05). Multivariate logistic regression included the factors with *P* < 0.05 in binary logistic regression. According to the results, Hypertension, Grade I plaque, HbA1c, MPV, and Fib were independent predictors (Table 2). On the included variables, LASSO regressions were conducted to evaluate the selected predictors' reliability further. Five predictors were selected based on the optimal value (*λ* = 0.0629). Variables included hypertension, Grade I plaque, HbA1c, MPV, and Fib. Consistent with the independent predictors identified by multiple regression, the regression coefficients were 0.203, −0.242, 0.135, −0.167, and 0.106 (Figure 2).

**Table 1:** Results of binary logistic regression analysis of the clinical characteristics of patients in the case and control groups.

Variables	All patients (*n* = 227)	Case group (*n* = 132)	Control group (*n* = 95)	Odds ratio [95% CI]	*P*-value
Male	181	107	74	0.823 [0.429–1.579]	0.559
Hypertension	174	109	65	2.187 [1.172–4.082]	0.014*
Smoking	78	45	33	0.919 [0.558–1.693]	0.919
Drinking	47	26	21	0.864 [0.452–1.561]	0.659
DM	92	57	35	1.303 [0.759–2.237]	0.338
SCS	88	50	38	0.915 [0.533–1.570]	0.746
Grade I plaque	13	10	3	0.198 [0.053–0.739]	0.016*
Grade IV plaque	55	36	19	1.500 [0.797–2.822]	0.209
P-max ≥ 4 mm	87	53	34	1.204 [0.698–2.076]	0.505
PS ≥ 5.6 mm	114	72	42	1.514 [0.891–2.574]	0.125
HCT ≥ 0.4	112	63	49	0.857 [0.506–1.453]	0.567
NEUT%≥60%	117	71	46	1.240 [0.731–2.103]	0.425
LY%≥28.4%	116	67	49	0.968 [0.571–1.640]	0.903
MONO%≥7.4%	105	67	38	1.546 [0.907–2.637]	0.110
PLT ≥ 203*10^9^/L	112	61	51	0.741 [0.437–1.258]	0.267
MPV ≥ 9.2 fl	111	55	56	0.497 [0.291–0.850]	0.011*
Alb ≥ 41 g/L	107	59	48	0.791 [0.466–1.343]	0.386
Fib ≥ 3.0 g/L	106	70	36	1.850 [1.081–3.167]	0.025*
β_2_-MG ≥ 1.8 mg/L	113	69	44	1.269 [0.748–2.153]	0.376
TC ≥ 3.5 mmol/L	112	64	48	0.922 [0.544–1.562]	0.762
TG ≥ 1.16 mmol/L	113	68	45	1.181 [0.538–2.002]	0.538
HDL ≥ 1.0 mmol/L	95	52	43	0.786 [0.461–1.341]	0.377
LDL ≥ 1.97 mmol/L	113	61	52	0.710 [0.418–1.206]	0.206
Lp(a) ≥ 0.19 mg/dl	113	59	54	0.614 [0.361–1.044]	0.072
GFR ≥ 91 ml/min	110	63	95	0.932 [0.550–1.580]	0.795
Cr ≥ 71 μmol/L	113	68	45	1.181 [0.696–2.002]	0.538
Hcy ≥ 14 μmol/L	108	59	49	0.759 [0.447–1.288]	0.306
Age ≥ 70 years	57	34	23	1.086 [0.590–2.000]	0.791
BMI ≥ 24 kg/m2	146	82	64	0.794 [0.456–1.383]	0.416
HbA1c ≥ 7.0%	76	53	23	2.100 [1.171–3.767]	0.013*

SCS, symptomatic carotid stenosis; DM, diabetes mellitus; HbA1c, hemoglobin A1c; HCT, hematocrit; LY%, lymphocyte percentage; MONO%, monocyte percentage; PLT, platelet count; MPV, mean platelet volume; Alb, albumin; Fib, fibrinogen; TC, total cholesterol; TG, triglycerides; HDL, high density lipoprotein; LDL, low density lipoprotein; Lp(a), lipoprotein(a); GFR, glomerular filtration rate; Cr, creatinine; Hcy, homocysteine; PS, sum of the plaque thickness; P-max, maximum of the plaque thickness.

*Indicates statistically significant variables.

**Table 2:** Multiple logistic regression analysis results.

Variables	β	Odds ratio	95%CI	*P*-value
Hypertension	0.919	2.506	1.269–4.948	0.008*
Grade I plaque	−1.518	0.219	0.055–0.877	0.032*
MPV ≥ 9.2 fl	−0.783	0.457	0.258–0.811	0.007*
Fib ≥ 3.0 g/L	0.629	1.876	1.053–3.342	0.033*
HbA1c ≥ 7.0%	0.696	2.005	1.080–3.723	0.028*

MPV, mean platelet volume; Fib, fibrinogen; HbA1c, hemoglobin A1c.

*Indicates statistically significant variables.

### Nomogram's building and apparent performance

**Figure 3:**
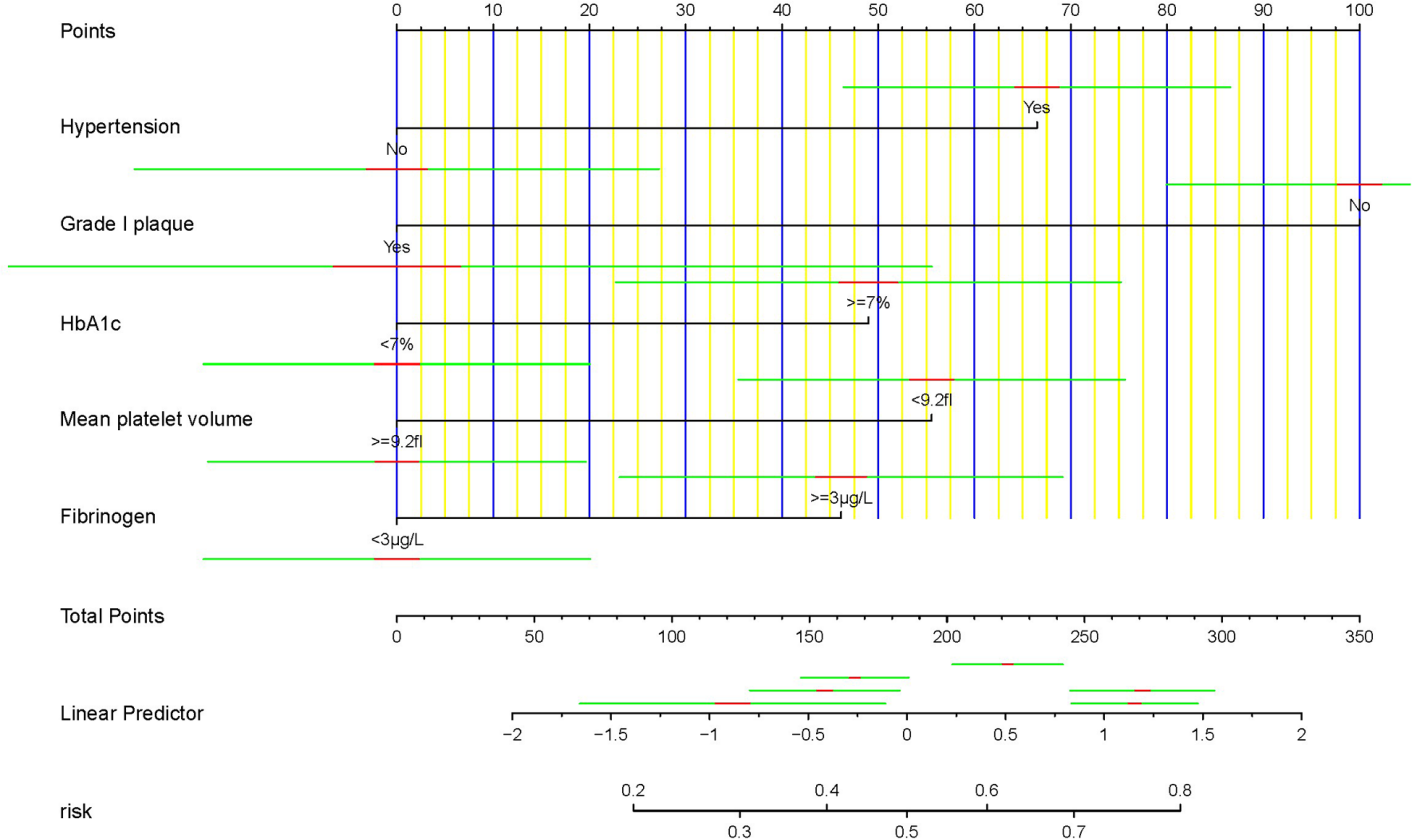
Nomogram for predicting the incidence of asymptomatic obstructive coronary stenosis. The construction of the nomogram included Hypertension, Grade I plaque, HbA1c, MPV, and Fib.

On the basis of the outcomes of logistic regression and LASSO regression, five predictors were eliminated and a nomogram risk prediction model was developed (Figure 3). Each variable was assigned a score, and the higher the score, the greater the risk of obstructive coronary stenosis in carotid stenosis patients.

### Assessment of the degree of calibration for the risk prediction model

**Figure 4:**
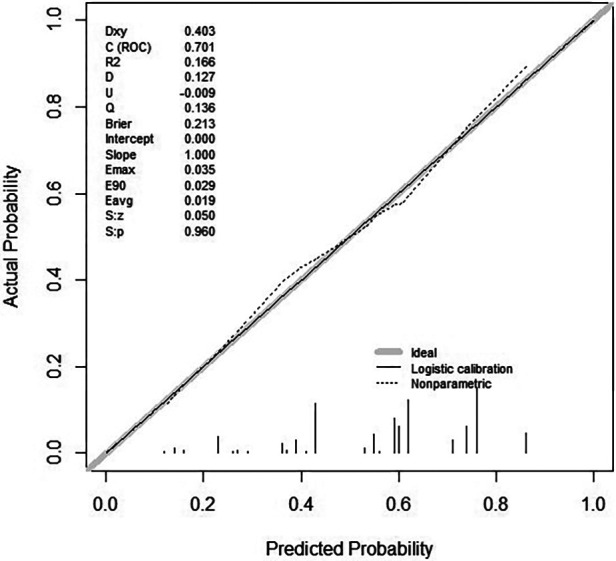
Curve of calibration for the risk prediction model of asymptomatic obstructive coronary stenosis.

To facilitate the evaluation of the risk prediction model's degree of rectification in this investigation, we developed a correction curve. As shown in Figure 4, the x-axis represents the predicted risk for asymptomatic obstructive coronary stenosis, and the y -axis represents the realization of asymptomatic obstructive coronary stenosis. The solid diagonal line represents the prediction of the ideal model, and the dashed line represents the actual predictive ability. The closer the dashed line is to the diagonal, the greater the predictive ability. Hosmer-Lemeshow test showed that the model had good calibration (*χ*^2^ = 2.24, *P *= 0.987).

### Assessment of the accuracy

**Figure 5:**
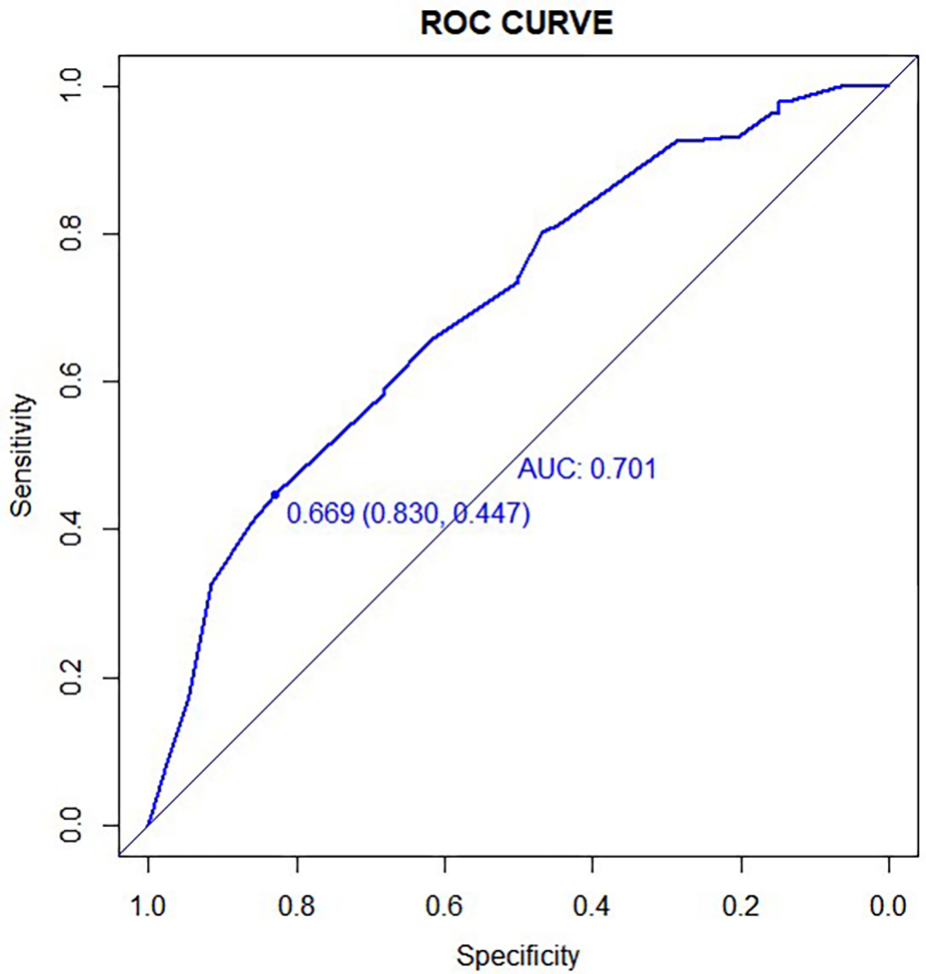
The ROC curve corresponding to the asymptomatic obstructive stenosis risk prediction model. The AUC value was 0.701%.

To evaluate the model's accuracy, we first calculated the C-index. When plotting a ROC curve, we can use AUC to quantify the predictor's efficacy. We then determined the model's AUC and constructed a ROC curve. The calculated AUC values of 0.701 (95% CI: 0.633–0.770) indicate that the model is accurate (Figure 5). The ROC curve was internally validated using the Bootstrap 1,000 times self-sampling method, and the AUC was found to be 0.668.

### Clinical net benefit

**Figure 6:**
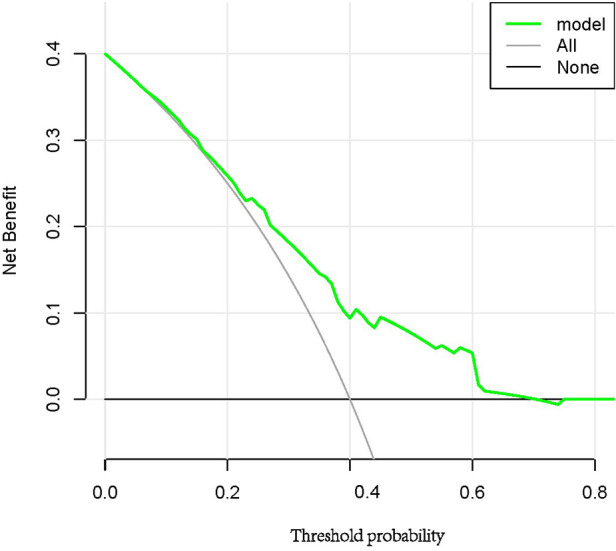
DCA for the asymptomatic obstructive coronary stenosis risk prediction model.

DCA was utilized to determine if the prediction model could enhance clinical decision-making (Figure 6). The y-axis indicates the net benefit, and the x-axis represents the probability of the threshold value. The black solid line represents no intervention, at which the net benefit is zero. The Grey solid line represents the intervention, and the net benefit is an oblique line with a negative slope. The green solid line represents the realized profits of the asymptomatic obstructive coronary stenosis risk prediction model. The DCA curve shows that the threshold of 0.08–0.70 has the largest clinical net income.

## Discussion

This study examined the statistical characteristics, imaging examination, and laboratory examination results of asymptomatic coronary artery stenosis in patients with carotid artery stenosis ≥ 50%. Univariate and multivariate logistic regression were utilized to identify independent risk factors, including hypertension, grade I plaque, HbA1c, MPV, and Fib. Using LASSO regression, the accuracy of the selected predictive factors was further confirmed, and a clinical predictive model was developed. According to the ROC curve, the AUC of the prediction model is 0.701, indicating that the model has a high degree of discrimination. Fit tests and clinical decision curves demonstrated that the predictive model was well calibrated and applicable in the clinical setting. Due to the limited sample size of this study, all data were only modeled as a training set, and the model was Bootstrapped 1,000 times for internal validation. The results demonstrate that the model of prediction is stable.

The most common cause of carotid stenosis is atherosclerosis, a multifactorial, progressive systemic disease (12). Plaque rupture, arterial embolism caused by thrombosis, and hemodynamic disturbance caused by stenosis comprise the majority of the carotid stenosis-induced ischemic stroke mechanism (13). Coronary artery disease is more common in carotid stenosis patients. According to studies, the prevalence of coronary artery disease in patients with carotid stenosis ranges from 13% to 86%, with the prevalence increasing with the severity of carotid stenosis (4). Asymptomatic coronary stenosis is easier to neglect than symptomatic coronary stenosis due to the absence of typical symptoms. Consistent with previous research, 58.1% of the 132 patients in this study experienced moderate or severe coronary stenosis complications. Due to the substantial increase in cardiovascular risk among patients with carotid artery stenosis and coronary artery stenosis coexisting, it is crucial to investigate the coexistence of carotid artery and coronary artery stenosis.

Hypertension was an independent risk factor for carotid stenosis with asymptomatic obstructive coronary stenosis in this study. High blood pressure damages the arterial wall and increases collagen deposition in the stroma, which causes the tunica media to thicken and the artery to become constricted. Hypertension is frequently viewed as a disease that damages large and small arteries, and lesions of corresponding arteries contribute to the occurrence and progression of hypertension. Prior research has demonstrated that hypertension is a risk factor for stroke. Patients with hypertension are substantially more susceptible to suffering a stroke than those with normal blood pressure (14). In a study of the lifetime risk of coronary heart disease, the lifetime risk of coronary heart disease in male hypertensive patients was 26.95% and the lifetime risk of coronary heart disease in female hypertensive patients was 14.85%, both of which were higher than the average level (15). High blood pressure is more likely to cause injury to the carotid and coronary arteries, resulting in their constriction.

HbA1c is produced by a sluggish, continuous, and irreversible glycation reaction and may reflect changes in patients’ blood glucose levels over the previous 8 to 12 weeks. In clinical practice, it is considered an essential indicator of blood sugar control in diabetic patients. Studies have shown that HbA1c levels are strongly associated with the risk of diabetes-related complications and cardiovascular disease (16). In general, a glycated hemoglobin level below 7.0% is advised for glycemic control (17). In a study of healthy controls and subjects with T2DM, glycemic fluctuations rather than persistent glycemic elevation caused endothelial cell injury, and intensive glycemic control reduced the incidence of microvascular and macrovascular complications by 25% (18, 19). In this study, patients with HbA1c levels greater than 7.0% were more likely to have asymptomatic coronary stenosis (OR = 2.005). It is advantageous to reduce this risk by enhancing blood glucose regulation and decreasing blood glucose fluctuations.

The investigation included imaging measures to assess the morphology of the plaque. CEUS is a common noninvasive technique for detecting plaque neovascularization. New blood vessels in the plaque are immature and readily rupture, resulting in inflammation of the vessel wall and vessel wall injury (20). High-grade carotid plaques as measured by CEUS are associated with more severe and unstable coronary artery disease, and the carotid plaque enhancement intensity is an independent predictor of secondary cardiovascular events in patients with coronary heart disease (21). This study evaluated the coronary status of patients with grade I or IV plaque and discovered that patients with grade I plaque were substantially less likely to develop asymptomatic obstructive coronary stenosis than controls (OR = 0.219). Using carotid plaque grade as a predictive parameter may increase the probability of identifying coronary stenosis.

Fib is a coagulation factor that promotes platelet aggregation, the growth, proliferation, and contraction of smooth muscle and endothelial cells, as well as increases blood viscosity and damages endothelial cells. Consequently, it is crucial to the pathogenesis of cardiovascular disease. Studies have shown that high levels of Fib are associated with an increased risk of stroke and poor patient outcomes (22). In terms of coronary stenosis, elevated Fib levels are generally regarded as an independent predictor of the presence and severity of coronary artery disease. This may be because fibrinogen stimulates the migration and proliferation of smooth muscle cells, and its degradation products are chemotactic for leukocytes and macrophages (23, 24).

MPV ≥ 9.2fl was a predictor of coronary stenosis events (OR = 0.457) and was protective in this study. MPV reflects the average platelet size, and aberrant MPV is associated with a variety of diseases, including diabetes, cardiovascular disease, and chronic obstructive pulmonary disease (25). High MPV levels have been associated with ST-segment elevation myocardial infarction in patients with coronary heart disease, and it is also regarded as an independent risk factor for inadequate coronary collateral compensation and a poor prognosis (26, 27). The above conclusion differs somewhat from the findings of this study, likely because the majority of the previous studies focused on the effects of acute coronary artery disease. In a study examining the effect of MPV on long-term outcomes following coronary intervention in patients with stable coronary artery disease, decreased MPV levels were associated with inferior outcomes (28). In addition, studies have demonstrated a correlation between systemic inflammation and platelet size. The MPV decreases as the degree of inflammation increases, whereas the MPV increases as the degree of inflammation decreases (29). The aforementioned findings suggest that elevated MPV does not increase the risk of stable coronary artery disease in patients with carotid artery stenosis; however, additional research is required to determine its cardiovascular effects.

This study analyzed the risk factors for asymptomatic obstructive coronary artery stenosis in patients with carotid artery stenosis ≥ 50% and constructed a clinical model with strong predictive ability. Nevertheless, given that this is a retrospective investigation, there are some limitations. First, it is necessary to exclude cases with insufficient data, which could introduce selection bias, and additional prospective studies are required for validation. Secondly, the sample size of this study is insufficient, and the proportion of male patients is high; therefore, it is necessary to increase the sample size in order to further assess the results' reliability.

## Conclusion

This study concluded that there is a significant correlation between carotid artery stenosis and coronary artery stenosis, and that asymptomatic and occult coronary artery stenosis warrants special attention. In patients with carotid stenosis, hypertension, grade I plaque, mean platelet volume ≥ 9.2 fl, fibrinogen ≥ 3.0 g/L, and glycosylated hemoglobin ≥ 7.0% were independent predictors of asymptomatic obstructive coronary stenosis. The clinical diagnostic model based on the aforementioned variables is capable of predicting the risk of coronary artery stenosis to a certain degree.

## Data Availability

The raw data supporting the conclusions of this article will be made available by the authors, without undue reservation.
